# Time course of cardiovascular responses to acute sustained handgrip exercise in young physically active men

**DOI:** 10.14814/phy2.70286

**Published:** 2025-04-02

**Authors:** Vladimir N. Melnikov, Tamara G. Komlyagina, Valentina V. Gultyaeva, Dmitriy Y. Uryumtsev, Margarita I. Zinchenko, Ekaterina A. Bryzgalova, Irina V. Karmakulova, Sergey G. Krivoschekov

**Affiliations:** ^1^ Scientific Research Institute of Neurosciences and Medicine Novosibirsk Russia

**Keywords:** arterial stiffness, body composition, hand grip, inotropy, lusitropy, Sphygmocor

## Abstract

We aimed to assess currently unexplained effects of isometric exercise on central hemodynamic, arterial, and cardiac cycle parameters. Twenty‐three young physically active males performed 5‐min forearm sustained exercise at 20% of maximum voluntary contraction. The pulse wave analysis (SphygmoCor) was conducted at baseline (BL) and at 1, 5, 10, 15, and 20 min of post‐load recovery. The General Linear Model repeated measures analysis with post hoc test was used to compare the BL values, 1‐min, and 15‐min recovery states. Exercise immediately elevated central and peripheral systolic blood pressure (BP), augmentation index, left ventricular contractility, and its relative relaxation time. These prompt reactions were followed by a hypotensive response and positive lusitropic effect with shortening relaxation in 15 min after the contraction ceased. The diastolic BP decrement was inversely correlated with the amount of body lean mass and body muscle but not fat mass measured by the bioelectrical impedance method. It is hypothesized that (1) the body lean mass‐dependent BP‐lowering effect of exercise is due to the arterial distending influence of metabolites accumulated in the muscle during exercise‐induced occlusion and then washed out into general circulation, and (2) muscle arteries are more sensitive to these effects than vessels of fat tissue.

## 1 INTRODUCTION

Regular isometric exercise (IE) is now widely used as an integral component of training programs in sport and medicine for improving muscle performance and promoting health (Chrysant, 2010; Garber et al., 2011). It elicits a blood pressure (BP)‐lowering effect (Carlson et al., 2014; McGowan et al., 2017; Millar et al., 2014; Wiley et al., 1992) and could confer more benefits than dynamic exercise to hypertensive persons (Pagonas et al., 2017) or individuals having mobility problems or joint disorders.

An acute static exercise shortly increases the blood pressure (Ferguson & Brown, 1997; Hartog et al., 2018) and sympathetic nerve activity (Mark et al., 1985; Saito et al., 1990) through the signals from the motor cortex and the exercise pressor reflex (Mitchell et al., 1983). The latter reflex comprises mechanical and chemical skeletal muscle receptors and is generated by sustaining contraction if the muscle fatigues. This kind of load increases BP in normotensive individuals also due to adrenal activation via circulating norepinephrine and epinephrine and enhances cardiac performance and contractility (Gonzalez et al., 1989). Isometrically contracted skeletal muscle begins to use anaerobically obtained energy because of the insufficiency of oxygen delivery when the blood supply does not match metabolic demand, as in the case of intensive sustained exercise (Seals & Enoka, 1989; Smith et al., 2006) usually associated with muscle hypoperfusion or local working ischemia (Sadamoto et al., 1983).

A single bout of low‐intensity isometric handgrip (IHG) exercise later reduces blood pressure in pre‐ and hypertensive individuals (Millar et al., 2011; Olher et al., 2020). However, this effect cannot be considered a general phenomenon since Ash et al. (2017) did not support the using of acute or chronic IHG as hypotensive therapy in hypertensive patients. Although the hypotensive effect of physical training, including repeated static exercise, is a topic of many studies (Kounoupis et al., 2020; McGowan et al., 2017), the majority of works was carried out on cardiovascular (CV) patients (see Kelley & Kelley, 2010; Owen et al., 2010; Cornelissen et al., 2011 for reviews). However, several researchers reported that isometric training did not reduce BP (Pagonas et al., 2017; Seidel et al., 2021). Thus, data on prompt and postponed CV effects of IE of both acute and repeated modalities are quite inconsistent. Meanwhile, such data seem to be important for the correct understanding of physiological mechanisms of long‐term consequences and benefits of IE training.

Numerous data concern the effects of static exercise on arterial stiffness. Several studies have found a decrease in large artery distensibility in response to IHG exercise in healthy individuals (Geleris et al., 2004; Lydakis et al., 2008) suggesting that this decrease can be due to increased vasoconstriction from the activated sympathetic system. An enhanced aortic PWV, the recognized marker of increased arterial stiffness, has been found during (Reid & Conway, 2006) and shortly after the 30‐s maximal contraction in healthy persons (Hartog et al., 2018) and in patients with coronary artery disease (Moon et al., 2015).

In the majority of heart pathologies, myocardial relaxation is impaired (Kapelko, 2019; Marston & Pinto, 2023). The appropriate lusitropy is essential for shortening the heartbeat when the heart rate increases. Current literature suggests that lusitropy arises from protein kinase phosphorylation of troponin and phospholamban and subsequent speeding up of calcium intracellular transportation. While the biochemical mechanism of lusitropy is well understood, the cardiac relaxation rate is not commonly recorded and analyzed.

To the best of the authors' knowledge, only one paper describes the timing parameters of the cardiac cycle during exercise. Thus, Stock and colleagues (2020), having studied the profile of the aortic backward pressure wave in young adults at the end of the bout, found that acute HG exercise increased reflection magnitude and decreases the wave transit time and thus indicated the immediate arterial stiffening effect. As for the influences of exercise on the timing of left ventricular (LV) relaxation, no data are available in the literature.

The objective of the present study was to ascertain CV reactions, including phases of the cardiac cycle, to single HG exercise in healthy physically active male individuals and to verify factors, including habitual physical activity and body composition, upon which the responses depend. Our hypothesis was that a single session of isometric exercise exerts a delayed BP‐lowering effect during post‐exercise recovery probably due to the release of under‐oxidized vasoactive substances from the ischemized muscle and metabolic blood acidification. Based on the assumption of the metabolic mechanism of the exercise hypotensive effect, an additional hypothesis was tested that this effect depends on total vascular bed size, that is, body mass, mass segmental distribution, and tissue composition. Since the arterial compliance substantially determines the cardiac work via peripheral vascular resistance, myocardial afterload, and the reflection wave, characteristics of arterial elasticity and the returned wave were included in the set of the parameters measured.

## 2 METHODS

### 2.1 Subjects

Twenty‐three physically active male university students aged between 18 and 23 years were recruited in this interventional repeated measures study. Participants regularly trained at least 6 h a week in a swimming pool or performing exercises in a fitness hall or outdoors with running or skiing.

The Short Form of International Physical Activity Questionnaire (IPAQ) was used for measuring weekly physical activity (PA) (Craig et al., 2003), which was calculated as the sum of vigorous‐intensity, moderate‐intensity activities, and walking.

Subjects were asked to refrain from alcoholic and caffeinated beverage consumption and not to change their eating habits and usual sleep hours before the day of the experiment. All were apparently healthy and free of metabolic and cardiovascular disorders.

### 2.2 Protocol

**FIGURE 1:**
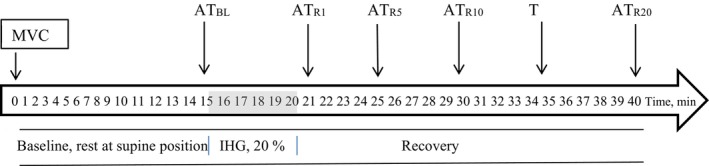
Study protocol. IHG, isometric hand grip exercise; MVC, maximum voluntary contraction; R1–R20, recovery at 1 min to 20 min. Arrows indicate applanation tonometry (AT) measurements.

The present study was carried out in autumn–winter season. Figure 1 shows the time protocol of the experiment.

### 2.3 Exercise stress test

The test was carried out in the morning (8:30–11:30). A spring hand dynamometer was used to assess maximum voluntary contraction (MVC) force in kg. After a rest period of 15 min, the subjects performed a 5‐min single sustained exercise bout with the right hand at 20% of MVC with visual feedback in a supine position. Basing on the assumption that exercise duration substantially determines CV responses, we have chosen this prolonged bout of 5 min that has not yet been investigated elsewhere. Though not by design, we have preliminarily tested several exercise protocols and chosen the minimal load intensity and duration that were quite fatiguing to alter the physiological parameters of interest. Furthermore, basing on the results of preliminary testing and supposing the duration of post‐exercise rest as a major determinant of the hemodynamic recovery, we have chosen the 20‐min rest period to assess the postponed dynamics of the parameters under study.

### 2.4 Applanation tonometry and pulse wave analysis

**FIGURE 2:**
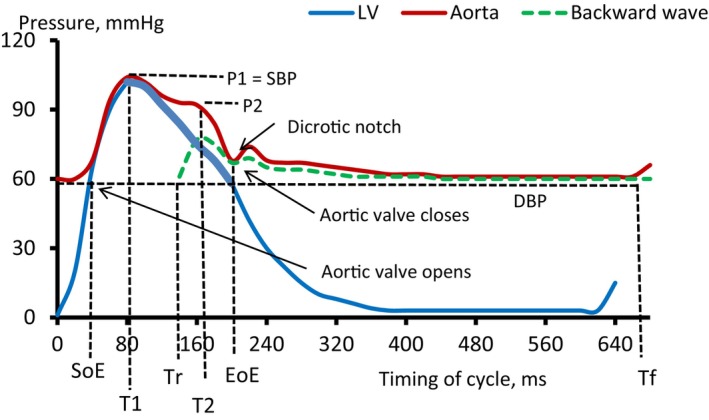
Aortic and LV pressure profiles and timing of the cardiac cycle in healthy young individuals (withdrawn from Wiggers' diagram, modified). SBP, DBP, systolic and diastolic blood pressure; EoE, end of ejection; P1, pressure at T1; P2, pressure at T2; SoE, start of ejection, the zero‐point for aortic cycle; T1, time to first peak; T2, time to second peak; Tf, end of cycle; Tr, time to the beginning of reflection. The x‐axis is distended for better visualizing time points. The bold curve corresponds to period of early (asynchronic) LV relaxation. T1 point represents the first peak of pulse wave that corresponds to maximum pressure of antegrade (forward) wave generated by LV and represents the duration of LV late contraction. The second systolic peak P2 (and T2) represents the maximum of reflection (backward) wave, coming to the aortic root at Tr, from the periphery. The P2 value is equal to the sum of the amplitudes of primary and reflected waves. The time difference between the opening and closing of aortic valve is ejection duration (ED = DoE–SoE). The difference between the cycle duration Tf and the end of ejection EoE corresponds to the duration of diastole (DD). The augmentation pressure (AugP, mmHg) is the difference between the amplitudes of the second and first systolic peaks. The augmentation index (AugI, %), adjusted to HR = 75, is calculated according to the formula (P2–P1)/(SBP–DBP) × 100 and corresponds to the proportion of AugP in pulse pressure. The Buckberg's Subendocardial Viability Ratio (SEVR) is the diastolic‐to‐systolic integral ratio and characterizes the myocardial perfusion, that is, oxygen supply/demand balance.

The assessment of central hemodynamics and arterial compliance was performed using the SphygmoCor device (AtCor Medical, Australia) with subjects in a supine position. After recording the blood pressure in triplicate in the left brachial artery using an automated oscillometric method (Omron M2 Basic, Omron HealthCare Co., Japan), the applanation tonometer was placed on the skin above the left radial artery at the wrist, and the peripheral pulse wave profile was continuously recorded by the apparatus. On the basis of experimentally established transfer function, the device algorithm then calculated features of the pulse wave in the aortic root (Figure 2).

Figure 2 schematically shows the normal aortic and LV pressure profile in young subjects. In addition to the set of parameters of PWA calculated by the device, we generated two new indices. Among them, there was the rise in aortic pressure value calculated as P1‐to‐(T1–SoE) ratio (i.e., dP/dT, mmHg/ms) that characterizes the rate, or “energetics”, of LV late contraction. Also, according to the recently published method (Melnikov, 2021) we assessed the relative value of early LV relaxation time (LVRT, %) as (EoE – T1)/Tf × 100 and considered it a surrogate characteristic of the cardiac lusitropic effect of the exercise.

### 2.5 Anthropometric measures and body composition

The absolute and relative fat and lean masses of the whole body and of its anatomical compartments were measured by the bio‐impedance method using the InBody‐370 device (InBody Co., Korea). Since no significant differences in right and left extremities could be found for correlations between somatometric and physiological variables, the recordings of the right limbs were included in the analysis. The body weight (BW), body mass index (BMI), body fat mass, body lean mass, trunk fat mass, arm lean mass, and leg lean mass were measured and expressed in percentage of BW where appropriate.

### 2.6 Statistical analysis

The general linear model repeated measures analysis was conducted by using SPSS‐19 software (SPSS Inc., Chicago IL, USA) to evaluate whether there was a difference in physiological variables over time after IHG load. Where a significant main effect was observed, then pairwise comparisons using post hoc Bonferroni adjustment were used to examine differences between resting and post‐exercise values. Among the latter, two time points were selected from five for statistical analysis, 1 min and 15 min of recovery, as the characteristic points for mostly pronounced positive and negative BP responses to exercise. Adjustment for anthropometric variables was performed by inserting them in the model as covariates.

The linear associations between physiological and other variables were evaluated by using partial correlation analysis with controlling for the effects of baseline HR. The Student's *t*‐test was used for comparing two subsamples of the studied population.

The two‐sided *p*‐value ≤0.05 was considered significant; however, the levels 0.05 < *p* ≤ 0.1 were also indicated as an evidence of a tendency in a difference, effect, or association. Descriptive statistics for the continuous variables are reported as means and standard deviations (M ± SD).

## 3 RESULTS

### 3.1 Participant's characteristics

Table 1 depicts the descriptive characteristics of the study population.

**TABLE 1:** Descriptive characteristics of subjects, *n* = 23.

Parameter	M ± SD (range)
Age, years	(18–23)
Body weight (BW), kg	74.3 ± 13.9
BMI, kg/m^2^	23.5 ± 4.1
Body muscle, % BW	36.0 ± 5.5
Body fat, % BW	13.0 ± 8.0
Height, cm	178 ± 5.6
Lean leg mass, % BW	9.5 ± 1.1
Total physical activity (IPAQ), MET‐minutes/week	4580 ± 2071
Maximum contraction strength, kg	43.5 ± 6.0

The vigorous activity demonstrated the direct association with lean mass on the extremities (*r* = 0.5–0.6, *p* < 0.01) and tended to negatively correlate with the relative amount of total fat mass and that on the trunk and extremities (*r* = −0.38–0.44, *p* < 0.1). As for the physiological parameters, two beneficial effects of high PA were established by correlation analysis. First, there was a weak association (*r* = 0.46, *p* = 0.043, HR = const) between the P1/T1 ratio at baseline and vigorous‐intensity activity. Second, the higher the walking activity, the more the Tr (*r* = 0.76, *p* < 0.001), that is, the later the backward pressure wave returns to the proximal aorta. Hence, according to the suggestion by Stock and colleagues (2020), the latter result can be considered as an evidence of compliant peripheral arteries in individuals with high PA. No correlations were found of PA with either baseline BP or its responses to exercise.

### 3.2 Physiological responses

**FIGURE 3:**
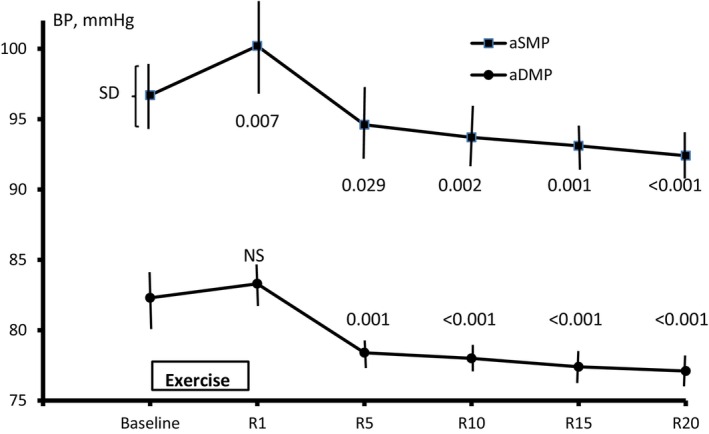
The time course of aortic systolic mean pressure (aSMP) and diastolic mean pressure (aDMP) before and after a 5‐min IHG single exercise bout at 20% of MVC. R, recovery, min. *p* value versus baseline (GLM repeated measures analysis with Bonferroni correction).

Figure 3 depicts average changes in central mean blood pressure values over the bout of examination. Immediately after the exercise, the SMP, but not the diastolic one, increased and both substantially fell thereafter below the pre‐exercise values.

Table 2 presents the results of a 3‐point repeated measures analysis (GLM) of physiological parameters in the course of recovery.

**TABLE 2:** Parameters of central hemodynamics, arterial elasticity, and timing of the cardiac cycle before and after sustained HG exercise (20% of MVC, 5 min) in young male individuals (GLM repeated measures, M ± SD, *n* = 23).

Parameter	Baseline (1)	Recovery 1 min (2)	Recovery 15 min (3)	*р* ^^^	*p* 1–2*	*p* 1–3*	*p* 2–3*	*η* ^2^
HR, beat/min	58.6 ± 9.1	57.9 ± 9.9	56.1 ± 9.0	0.065	NS	NS	NS	0.12
bSBP, mmHg	125.2 ± 8.1	130.9 ± 9.7	123.1 ± 8.2	<0.001	0.004	0.051	<0.001	0.46
bDBP, mmHg	70.1 ± 6.7	70.1 ± 6.2	65.4 ± 6.4	<0.041	NS	<0.001	<0.001	0.39
aSBP, mmHg	104.4 ± 6.5	108.3 ± 8.4	101.5 ± 7.0	<0.001	0.022	0.012	<0.001	0.46
аDBP, mmHg	71.2 ± 6.9	71.1 ± 6.3	66.1 ± 6.5	0.012	NS	<0.001	<0.001	0.41
aSMP, mmHg	96.6 ± 6.4	100.2 ± 7.4	93.1 ± 6.4	<0.001	0.020	0.003	<0.001	0.52
aDMP, mmHg	82.3 ± 6.4	83.3 ± 6.9	77.4 ± 6.4	<0.001	NS	0.001	<0.001	0.44
AugP, mmHg	−0.17 ± 2.85	1.39 ± 3.39	−0.65 ± 3.56	0.008	0.031	NS	0.012	0.20
AugI75, %	−8.9 ± 6.3	−5.1 ± 8.3	−11.0 ± 9.4	0.005	0.028	NS	0.012	0.22
LVRT, %	19.9 ± 2.9	21.4 ± 3.4	19.4 ± 3.3	0.001	0.001	NS	0.007	0.29
T1, %	11.6 ± 2.8	10.5 ± 2.0	11.4 ± 2.1	0.022	0.015	NS	NS	0.16
Р1/Т1, mmHg/ms	0.281 ± 0.073	0.331 ± 0.073	0.287 ± 0.059	<0.001	0.001	NS	0.007	0.31

Abbreviations: *η* ^2^, partial eta squared; a, aortic; AugI, augmentation index; AugP, augmentation pressure; b, brachial; DBP, diastolic blood pressure; DMP, diastolic mean pressure; HR, heart rate; LVRT, early left ventricular relaxation time; NS, nonsignificant; P1/T1, rate of pressure elevation in proximal aorta from diastolic value to primary maximum due to LV contraction; SBP, systolic blood pressure; SMP, systolic mean pressure; T1, time to first peak (maximum of antegrade wave).

^^^ *p* Value for trend.

* *p* Values for paired comparisons, Bonferroni post hoc test. Presented are HR and parameters demonstrating a significant timing effect.

No significant effect of exercise over time could be observed for HR, pulse pressure amplification, ejection duration, diastolic duration, time to reflected wave Tr and SEVR.

Systolic pressure values, AugI, LVRT, and P1/T1 increased from baseline shortly after the exercise (1 min) and then decreased at the R15 state versus both baseline and R1 states. Fifteen minutes after the contraction ceased, the group mean brachial and central DBP dropped more markedly below the pre‐exercised value than the systolic pressure.

According to the exercise effect respective to BL, the variables of interest are arranged in the following order from maximum to minimum eta‐squared, that is, the percent of total variation explained by the main factor: aSMP, aSBP, bSBP, aDMP, aDBP, bDBP, P1/T1, LVRT, AugI, AugP, and T1. Consequently, the systolic blood pressure values reveal the highest changes in response to HG across the afterload recovery, whereas the HR, AugP, and T1 show the low alterations. A comparison of the P1‐to‐T1 ratio (*η* ^2^ = 0.31) with HR (*η* ^2^ = 0.12) shows that the exercise exerts a more marked inotropic than chronotropic effect on the heart.

### 3.3 The physiological‐somatometric associations (Table 3)

**TABLE 3:** Coefficients of bivariate Pearson's correlation between the magnitude of physiological response to IHG exercise (∆Variable), body weight, and the somatometric traits.

Parameter	BW	BMI	Body muscle	Body fat	Trunk fat	Trunk lean mass	Right arm lean mass	Right leg lean mass
∆ P1/T1_R1–BL_	−0.38^	−0.37^		−0.39^	−0.40^			
∆ bDBP_R1–BL_	0.55**	0.47*	0.43*	0.42^	0.45*	0.39^	0.37^	0.43*
∆ aDBP_R1–BL_	0.54**	0.42^	0.45*	0.37^	0.41^	0.42^	0.40^	0.47*
∆ bSBP_R15–BL_						−0.40^	−0.42*	
∆ bDBP_R15–BL_	−0.43*		−0.43*			−0.41^	−0.39^	−0.54**
∆ aSBP_R15–BL_								−0.43*
∆ aDBP_R15–BL_	−0.42*		−0.44*			−0.42*	−0.40^	−0.55**
∆ SMP_R15–BL_	−0.39^		−0.39^			−0.42*	−0.38^	−0.47*
∆ DMP_R15–BL_	−0.39^		−0.38^				−0.36^	−0.47*

*Note*: Presented are only correlations with *p* ≤ 0.1.

^*p* ≤ 0.1, *p ≤ 0.05, **p ≤ 0.01.

The next step of the analysis was to answer a question of whether the extent and direction of physiological responses to exercise depend on the body composition. We hypothesized that, according to the assumed metabolic mechanism of the post‐exercise hypotensive response, the degree of the pressure reaction depends on the size of the body, that is, the volume of the vascular bed and hence the concentration of dilutive metabolites washed out from the ischemized muscle after the contraction ceased.

The effects of exercise on aDBP levels, considered in the dynamic concern, remained significant if BW, leg lean mass, and body muscle were inserted in the GLM repeated measures model as single covariates, with *p* values 0.032, 0.014, and 0.024, respectively, in comparison with *p* = 0.012 for the model without covariates. The correspondent *p* values for the aSMP level were 0.056, 0.056, and 0.068 as compared to *p* < 0.001 for the model without covariates. Thus, the main time effect under study remained significant for aDBP and tended to be significant for aSMP after adjustment for the somatometric parameters.

Although in the total sample no significant differences between BL and R1 states could be found in both brachial and aortic DBP values (Table 2), however, the response magnitude exhibited significant moderate positive correlations with all anthropometric traits (Table 3): the corpulent individuals shortly respond to exercise by the higher magnitude of diastolic pressure reaction than the slim persons. Thus, while the individuals from the subsample with BW < Median (*n* = 12) demonstrated a fall of average aDBP from BL to R1 (∆ = −2.00 ± 6.45 mmHg), the subjects having BW> = Me (*n* = 11) showed an increment (∆ = 2.40 ± 2.41 mmHg, *p* = 0.037, *t*‐test). The correspondent changes for bDBP values were −1.92 ± 6.45 and 2.30 ± 2.58 mmHg, respectively (*p* = 0.047).

In contrast to the positive associations of all anthropometric variables with both central and peripheral DBP changes during the first minute of post‐exercise rest, the amplitude of P1/T1 increment, that is, the rate of late LV contraction, in response to the exercise tended to inversely correlate with somatometric traits, characterizing the body fatness.

Obvious remarkable direct correlations were found in our population for the intensity of the performed exercise (HG strength in kg) with relative amounts of body muscle (*r* = 0.53, *p* = 0.010), trunk lean mass (*r* = 0.55, *p* = 0.007), and arm lean mass (*r* = 0.57, *p* = 0.005), but not with the physiological parameters measured at baseline and at both states of recovery.

As for the late DBP response at 15 min after exercise, the negative associations were found with the characteristics of lean mass. It is worth noting that the moduli of correlation coefficients for the leg lean mass were higher than those for the arm lean mass. Among the physiological parameters, the P1/T1 ratio at baseline demonstrated a weak correlation with vigorous‐intensity activity (*r* = 0.46, *p* = 0.043, HR = const) and Tr was reliably associated with walking activity (*r* = 0.76, *p* < 0.001).

## 4 DISCUSSION

We demonstrated in this study that a single session of isometric HG exercise revealed a two‐phase hemodynamic response at least over the 20‐min recovery. The first phase constitutes a hypertensive reaction, arterial stiffening, an increase in LV relaxation time, and contractility. Conversely, the second phase consists of a decrease in BP levels, presumably diastolic ones. This phase seems not to be a simple return of the pressure just due to mechanical compensation but is an active process because the BP value notably falls below the BL value.

At 1 min after contraction, the SBP and AugP values elevated compared to BL due to the known pressor reflex generated by fatigued skeletal muscle and resulted in the activation of sympathetic nerve activity (Mitchell et al., 1983; Victor et al., 1995), an increase in peripheral resistance (Lewis et al., 1983), and positive cardiac chronotropic and inotropic effects (Umeda et al., 2015; Watanabe et al., 2014). Physical exercise, both dynamic (Kaur et al., 2015) and static (Goodwin et al., 1972; Hartog et al., 2018), has been reported to cause an intensity‐dependent increase in arterial BP, termed exercise pressor reflex, triggered by skeletal muscle afferents responding to both mechanical (mechanoreflex) and local metabolic (metaboreflex) stimuli (Boushel, 2010; Delaney et al., 2010). The latter reflex is generated by an increase in protons, potassium, phosphate, adenosine, and prostaglandin concentrations in venous blood that was observed shortly after IHG exercise (Ogata et al., 2008).

As it has been reported by Sadamoto et al. (1983), isometric contraction elevates local pressure to the vasculature and limits or even entirely occludes blood flow, leads to tissue ischemia, hence decreasing O_2_ delivery and oxidative metabolism. The increased intramuscular pressure and mechanical compression of the vascular bed within the muscle during static contraction has been suggested as one of the main contributors to responses of general circulation (MacDougall et al., 1985). Application of experimentally reproduced post‐exercise arterial occlusion of the isometrically contracted muscle with a cuff traps metabolites in the muscle, prevents their washing out into the circulation, and delays the recovery of load‐caused decreased oxygen supply to non‐exercised muscle. In the situation without external occlusion, the oxygen delivery to resting muscles quickly returns to baseline value due to metabolic vasodilation (Ogata et al., 2008).

The role of acidic metabolites in reproducing the post‐exercise hemodynamic effects is supported by the fact that acid‐sensing ion channels blockade attenuates pressor and sympathetic responses to skeletal muscle metaboreflex activation (Campos et al., 2019).

As for the postponed hemodynamic responses, the available data on the acute IHG exercise‐induced hypotension are inconsistent (Yamada et al., 2022). The BP‐lowering effect has been shown in individuals with hypertension (Millar et al., 2011; van Assche et al., 2017) but not in middle‐aged adults with prehypertension and obesity (Ash et al., 2017). Ogata et al. (2008) have found a substantial increase in mean BP immediately after the 2‐min IHG load performed at 40% MVC and a subsequent return of the pressure to the pre‐exercise BL value. Unlike the results of the current study, the experimental design with the short recovery period of 3 min, applied by the authors, could not reveal the post‐exercise hypotension.

The time‐ and BW‐dependency of BP responses to single static exercise, established in the current work, may in part explain the inconsistency of literary data and individual differences in vascular components of reaction to IHG exercise (Watanabe et al., 2014) that depends not only on intensity, duration of exercise and a mass of the muscle involved but also upon the duration of the post‐exercise rest. Either 20 s (Lydakis et al., 2008) or 2 min of IHG loads (Ogata et al., 2008), or 1 min of exercise at 50% MVC and subsequent 4‐min rest (Watanabe et al., 2014) seems to be insufficient to accumulate the sufficient amount of metabolites in the fatiguing muscle to elicit a dilative effect on systemic circulation, and the chosen recovery was too short to reveal hypotension. Our combination of strength and duration of the muscle contraction as well as the length of the post‐load rest occurred to be able to reveal such an effect.

The late post‐exercise systemic hypotension can be due to an accumulation of acid anions (Gaynullina et al., 2022). In the case of intensive static muscle contraction, the non‐oxidized metabolites, for example, protons (Pryor et al., 1990) can be accumulated because of muscle occlusion and under‐perfusion, with a resultant increase in anaerobic metabolism. Devereux with colleagues (2012) explored the physiological and biochemical consequences of 4‐week bilateral leg isometric exercise training and have reported a reduction in resting BP and related this effect with accumulation of lactate. Regarding the HR modulation, the lactate infusion decreases the cholinergic activity and augments sympathovagal ratio in healthy persons (Yeragani et al., 1994). Davis et al. (2020) have established a direct correlation among base deficit and blood lactate level in trauma patients following injury. The compressive trauma leads to hypotensive shock in a time‐dependent fashion after withdrawing occlusion because of the massive release of vasodilative metabolites into general circulation and requires infusing a base‐enriched solution for preventing the shock. The fatiguing isometric voluntary muscle contraction can be considered a fragile model of arterial occlusion occurring in cases of traumatic limb strangulation or compressing an extremity with a tourniquet.

The deferred cardiovascular beneficial effect of a single fatiguing isometric exercise, established in our study, can be accumulated since the IHG training exerts a long‐lasting BP‐lowering effect, exceeding that of dynamic exercise training, in prehypertensive (Cornelissen et al., 2011) or hypertensive (Pagonas et al., 2017) individuals.

As for the elasticity of conduit arteries, our findings suggest that IHG exercise promptly increases central AugI, that is, the relative amplitude of the reflected wave that is generally recognized as a valuable marker of arterial stiffness and is considered as such in many studies (Santos et al., 2021). This result is in line with the findings of other studies that have established a prompt elevation of AugI and PWV (Kalfon et al., 2015; Tanaka et al., 2014; Yao et al., 2017) via increasing sympathetic nerve activity (Victor et al., 1995). An earlier return of the reflected wave after a single isometric HG session, that is, the higher arterial stiffness, has been found in young adults (Stock et al., 2020) and healthy aging persons (Stock et al., 2023). In contrast to acute exercise, the isometric resistance training reduced PWV in hypertensive patients (Lopes et al., 2021), that is, made arteries more compliant. The 30 studies included in the meta‐analysis have shown that PWV decreased 30 min after the acute dynamic exercise due to decreased peripheral resistance, whereas it was not modified immediately after exercise (Saz‐Lara et al., 2021).

As for the LV contractility, in our work, the time of the first systolic peak of aortic pressure wave T1 was found to decrease compared to baseline. The fall in T1 was obviously associated with the rise in the P1‐to‐T1 ratio. This finding is in line with the results of Stefani and colleagues (2009) who have evaluated the Longitudinal Peak Systolic Strain as a measure of LV contractility by the echo imaging and Speckle Tracking analysis in athletes. They have demonstrated that an acute HG exercise at 30% of MVC shortly produced a ventricular deformation denoting an increase in LV contractility in athletes but not in sedentary controls.

Regarding the timing structure of a cardiac cycle under the influence of static exercise, to the best of our knowledge, only Stock with colleagues have explored the time of reflected wave (2020). We could not find changes in ED, DD, and reflection time (Tr). However, the LVRT promptly increased after the load that pointed at the transient negative lusitropic effect of static exercise.

The heart appears to sacrifice its “interests” in favor to the whole organism, fighting with an acute stressor, prolongs the ventricular relaxation, that is, experiences negative lusitropic effect, and hence worsens the LV filling. However, this temporary effect is compensated over post‐exercise rest, does not accumulate itself and does not become permanent in intensively training elite sportsmen. Thus, we have examined 13 sportsmen, presented in our laboratory's data base, of International Master's degree and found the similar LVRT compared with that of 31 age‐matched beginning sportsmen at rest (18.3 ± 5.4% vs. 17.9 ± 4.5%, respectively, *p* > 0.05).

In consistence to our results, the 20% load of MVC did not change SEVR, whereas the 70% exercise decreased it compared to BL (Lydakis et al., 2008). It seems likely that the exercise intensity is more significant determinant of subendocardial blood flow than the duration of the session.

With regard to physiological‐somatometric inter‐relations, the delayed hypotensive effect was found in the present study to depend on the body muscle mass but not fat mass. It should be noted that the coefficients of correlation between the post‐exercise DBP decrement and lean body mass, even if they are significant, are low, ranging between 0.35 and 0.55 and thereby account for a small portion of the observed variance of DBP. The fact that the hemodynamic response to isometric exercise depends on the muscle mass (Table 3) supports the suggestion in favor of the metabolic mechanism of delayed post‐exercise systemic BP‐lowering and arterial debilitating effects of HG single session demonstrated in the present study. Lewis and colleagues (1985) have described the role of muscle mass in determining the prompt hemodynamic response to isometric 6‐min contraction, comparing HG and two‐legged knee extension, and found that the pressor effect was related to the active muscle mass.

The heavier subjects increase their DBP immediately after the exercise, probably due to a more active pressor reflex, whereas the lean individuals decrease it. The plump subjects, who have a voluminous bloodstream, show a smaller decrease in blood pressure during 15 min of rest than thin individuals, since in the slim persons, acidic metabolites are distributed in a smaller vascular volume, reach greater blood concentration, and therefore exert a higher hypotensive effect.

The lack of correlations between the parameters of fatness, including BMI, and the magnitude of the late hemodynamic response to the exercise after 15 min of rest (Table 3) allows suggesting that resistant arteries in adipose tissue are less sensitive to dilative metabolites generated by isometrically contracting skeletal muscle than arteries of lean tissues.

The present work yielded a tendency of negative association between the incremental changes of late LV contraction rate and body adiposity. The P1/T1 gain from BL to R1 tended to be lower in heavier subjects having more fat mass without any associations with lean mass: the high relative amount of adipose tissue decreases the immediate positive inotropic effect of exercise. The findings of de Wit‐Verheggen with colleagues (2020) shed light on this association. The authors have found that a large amount of pericardial fat limits the myocardial distensibility and contributes to diastolic dysfunction. Probably, it is not the fat mass itself that determines the cardiac response, but other likely mechanisms can be responsible for this association, for example, biochemical characteristics of lipid metabolism. The observed weak inverse dependence of the positive inotropic effect of exercise upon fatness, if it is a real one, appears to be an important novel result of the present study and might suggest hypotheses for further investigations. One of the possible determinants might be sphingosine phosphate, a signaling sphingolipid that controls acute cardiac chronotropic and inotropic responses to stressors (Jozefczuk et al., 2020).

In consistence with existed numerous findings (Foong et al., 2014), our results show that high physical activity improves body composition, leading to fat mass reduction. Thus, the relative amount of total fat mass and that on the trunk and extremities tended to negatively correlate with vigorous PA. Moreover, two beneficial effects of high PA on physiological parameters were established by correlation analysis. First, a weak positive association was found with LV contractility measured by the P1‐to‐T1 ratio. Second, the higher is PA the more is the Tr, that is, the later the backward pressure wave returns to the proximal aorta. Hence, in accordance with the data by Stock et al. (2020), individuals with higher PA have compliant peripheral arteries. Unlike the current results of no association between PA levels and BP variables in young men, in a previous study performed on healthy individuals aged 20–59 years, we have reported an inverse correlation of habitual PA with BP levels at rest but not with pressure increment in immediate response to IHG exercise (Gultyaeva et al., 2019).

Interpretation of the results of the present study is limited by the following concerns. First, we did not measure intramuscular blood perfusion and can only suppose the occlusion of forearm circulation during exercise. However, the finding by Schnizer et al. (1979) on post‐exercise increase in forearm volume due to extravascular volume supports this supposition. Second, we did not measure the blood lactate level. Third, the applanation tonometry measures only the duration of the fast part of the descending curve of the aortic pulse wave and commensurately indirectly assesses the initial part only of the total LV relaxation. Further, given the nonsignificant correlations obtained with a *p* value ≤0.1, additional studies of larger samples are warranted to confirm the findings established on the association of hemodynamic variables with anthropometric traits. These limitations characterize the study as a pilot one. Further studies are needed to determine the cellular mechanisms driving the inotropic and lusitropic effects of muscle isometric contraction and blood acidification.

In conclusion, we demonstrated that time after a single static exhausting exercise is essential for the hemodynamic response. The load exerts a two‐phase cardiovascular effect. The immediate hypertensive response is rapidly followed by the BP‐lowering reaction. The prompt negative cardiac lusitropic effect and the arterial stiffening are replaced by subsequent accelerating LV relaxation and distending arterial walls. The delayed DBP decrement negatively correlates with body lean mass but not fat mass. This finding allows forwarding a hypothesis that arteries in adipose tissue are less sensitive to dilative metabolites generated by isometrically contracting skeletal muscle than arteries of lean tissues.

## AUTHOR CONTRIBUTIONS

VNM: methodology, data analysis, and writing original draft; TGK: investigation, applanation tonometry, and formal analysis; VVG: anthropometry, formal analysis, writing—review and editing; DYU: investigation, validation, and database managing; MIZ: investigation, medical inspection, and organizational support; EAB: literary searching and analysis; IVK: visualization, statistical analysis, and drawing figures; SGK: supervision and conceptualization. All authors read, revised the manuscript, and approved the submitted version.

## FUNDING INFORMATION

None.

## CONFLICT OF INTEREST STATEMENT

The authors declare that they have no conflicts of interest.

## ETHICS STATEMENT

All the procedures performed are in line with the Declaration of Helsinki and correspond to the ethical standards for studies involving human participants. The study protocol was approved by the Local Ethical Committee of the Institute of Neuroscience and Medicine.

## CONSENT TO PARTICIPATE

All subjects gave written informed consent to participate in the study.

## CONSENT TO PUBLICATION

Consent was obtained from all participants.

## Data Availability

The data supporting the findings of this study are available from the corresponding author upon reasonable request.
